# Dual-Energy Computed Tomography (DECT) Resolves the Diagnostic Dilemma in an Atypically Presenting Case of Gout

**DOI:** 10.7759/cureus.38247

**Published:** 2023-04-28

**Authors:** Nirali Sanghavi, Sindhuja Korem, Subo Dey, Amy Wasserman, Julia Ash

**Affiliations:** 1 Internal Medicine, Westchester Medical Center, Valhalla, USA; 2 Rheumatology, Westchester Medical Center, Valhalla, USA

**Keywords:** dect providing high quality images, gouty tophi, monosodium urate deposition, rheumatoid arthritis flare, gout flare

## Abstract

Gout is a common inflammatory arthropathy that presents as acute monoarthritis, most commonly of the first metatarsophalangeal (MTP) joint. Chronic polyarticular involvement may lead to confusion with other inflammatory arthropathies, including rheumatoid arthritis (RA). A thorough history, physical examination, synovial fluid analysis, and imaging are keys to establishing a correct diagnosis. Although a synovial fluid analysis remains the gold standard, the affected joints may be difficult to access by arthrocentesis. In cases where a large monosodium urate (MSU) crystal deposition is in the soft tissues - the ligaments, bursae, and tendons, it becomes a clinical impossibility. In such cases, dual-energy computed tomography (DECT) can assist in differentiating gout from other inflammatory arthropathies, including RA. Additionally, DECT can perform quantitative analysis of tophaceous deposits and, therefore, assess response to treatment.

## Introduction

Gout is a metabolic disease with a prevalence of 3.9% in the US [1]. The prevalence in men is 3-6% and in women, it is 1-2%. Prevalence also increases with age [1]. It presents as acute, usually monoarticular, arthritis, with an intercritical period, and chronic tophaceous gout [2]. However, without adequate treatment, it can lead to chronic polyarthritis, which may eventually mimic rheumatoid arthritis (RA) [2]. Differentiating polyarticular gout and RA can be extremely challenging, and it is also important to rule out their co-existence in the same patient that presents atypically{2}. We report a rare case of a patient with polyarticular gout with unusual manifestations like the absence of interictal period and podagra but with the involvement of multiple large joints. In addition, radiological imaging with signs of RA and uric acid deposition was seen mainly in ligaments and tendons on dual-energy computed tomography (DECT). As uncomplicated cases of gout can also be treated by primary care physicians, our case report highlights the awareness and utility of DECT in establishing an accurate diagnosis.

## Case presentation

A 50-year-old Hispanic man with a history of non-crystal-proven gout and type-2 diabetes mellitus presented to Westchester Medical Center Rheumatology Clinic with a chief complaint of persistent right wrist pain for the past six months. He also reported pain and swelling of the left wrist, bilateral ankles, and knees for the past eight years. The pain would recur every few weeks and last for seven days but lately become more constant. No arthrocentesis was performed in the past. History was negative for fever, chills, morning stiffness, and trauma. The patient did not smoke or drink alcohol and had no family history of gout or autoimmune diseases. He was treated by his primary care physician with allopurinol 300 mg daily, colchicine 0.6 mg daily, and indomethacin as needed for pain but had no relief of symptoms. Past serum uric acid levels ranged between 7 and 10 mg/dL (normal range 3.5-7.2 mg/dL) while on allopurinol treatment.

The musculoskeletal examination was significant for swelling, mild erythema, and tenderness of bilateral wrists, R>L, right fourth metacarpophalangeal (MCP), and right fifth proximal interphalangeal (PIP) joints. There was tenderness of the right knee without effusion and with flexion limited to 60 degrees and swelling of the first to fifth right MTP joints. There were no tophi, hand deformities, and skin rashes, including psoriasis. Respiratory, cardiovascular, and neurological examinations were unremarkable. Laboratory analysis was significant for elevated C-reactive protein (CRP) at 2 mg/dL (normal 0-0.5 mg/dL) and slightly elevated serum calcium at 10.3 mg/dL. Complete blood cell count, comprehensive metabolic panel, and intact PTH were normal. Serum Lyme enzyme-linked immunosorbent assay (ELISA), rheumatoid factor, and anti-CCP ab were negative. The serum uric acid level was 6.6 mg/dL.

**Figure 1:**
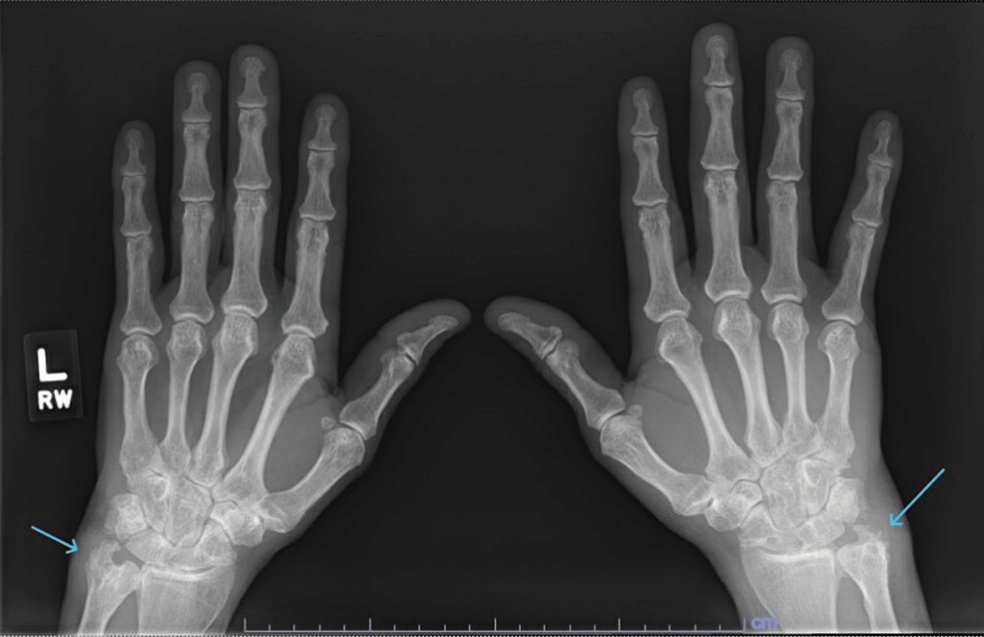
X-ray of hands showing increased bony proliferative changes, erosion, and soft tissue swelling at the ulnar styloids

**Figure 2:**
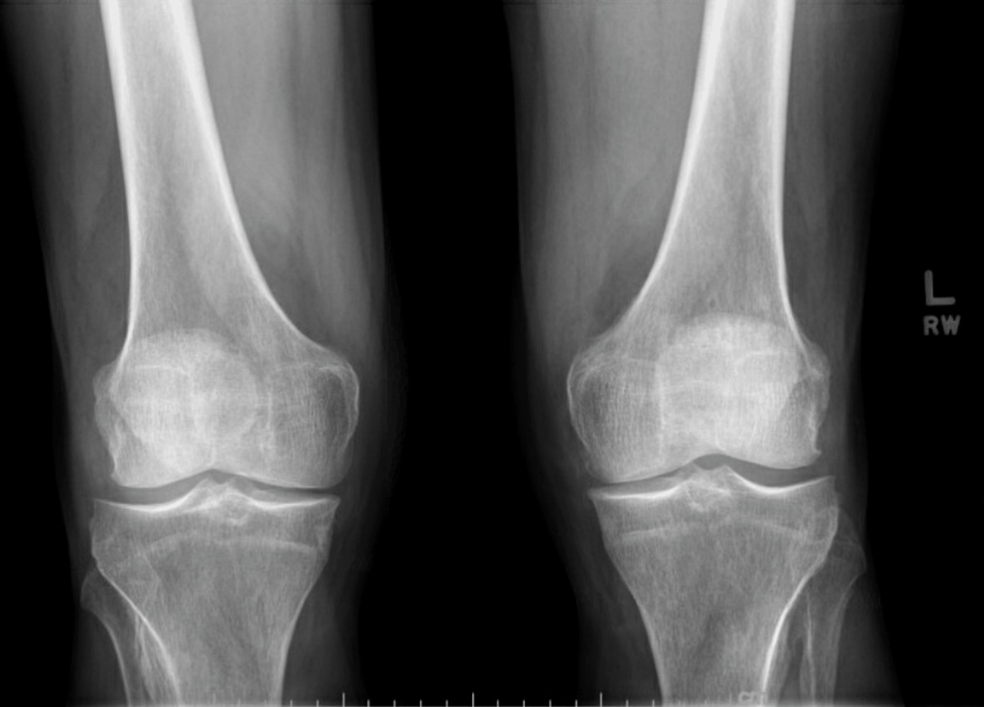
X-ray of bilateral knees demonstrating mild joint space narrowing, patellofemoral joint space narrowing, periarticular osteophytes, small suprapatellar effusion (right more than left)

**Figure 3:**
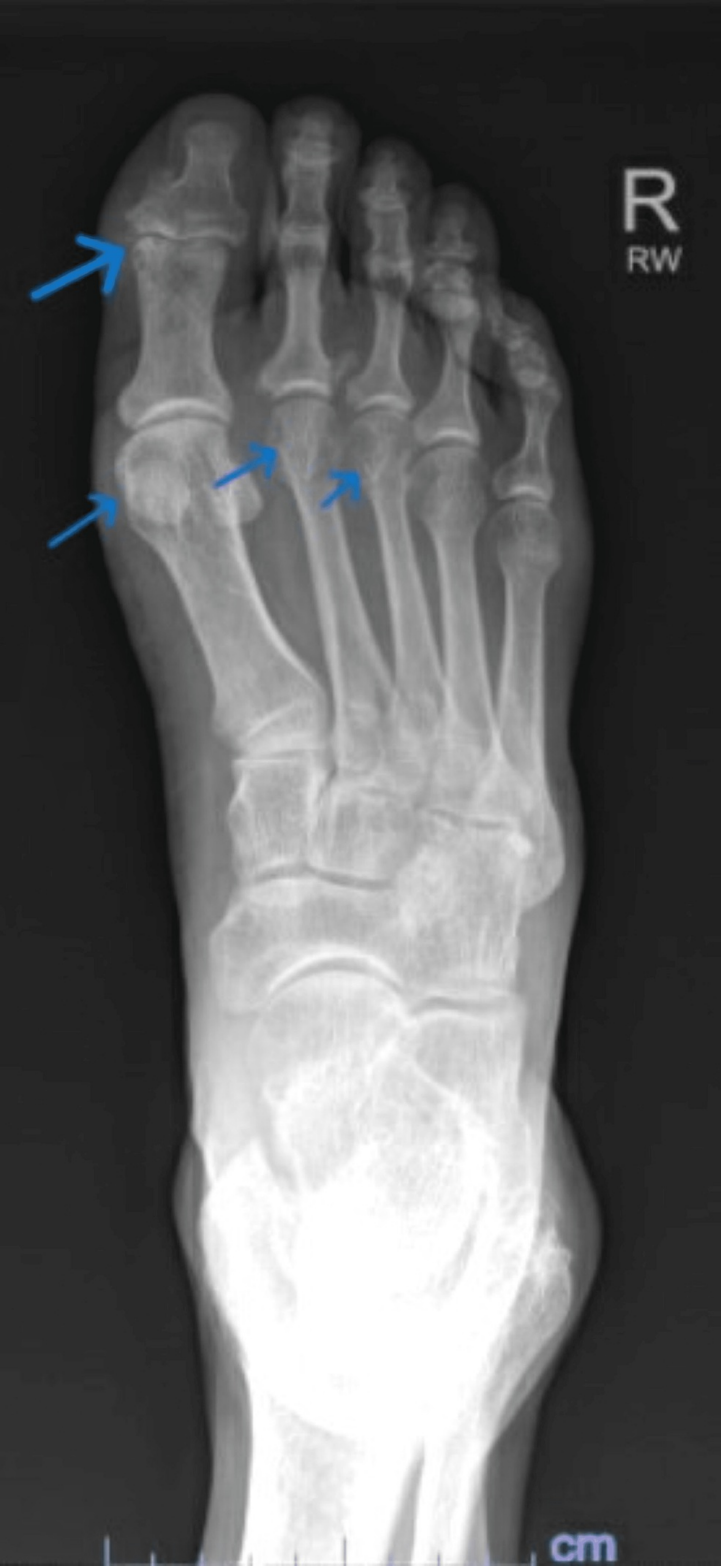
X-ray of the right foot revealing narrowing, sclerosis, and a subchondral cyst at the right first IP joint and erosions of the first, second, and third MTP joints IP: interphalangeal; MTP: metatarsophalangeal

**Figure 4:**
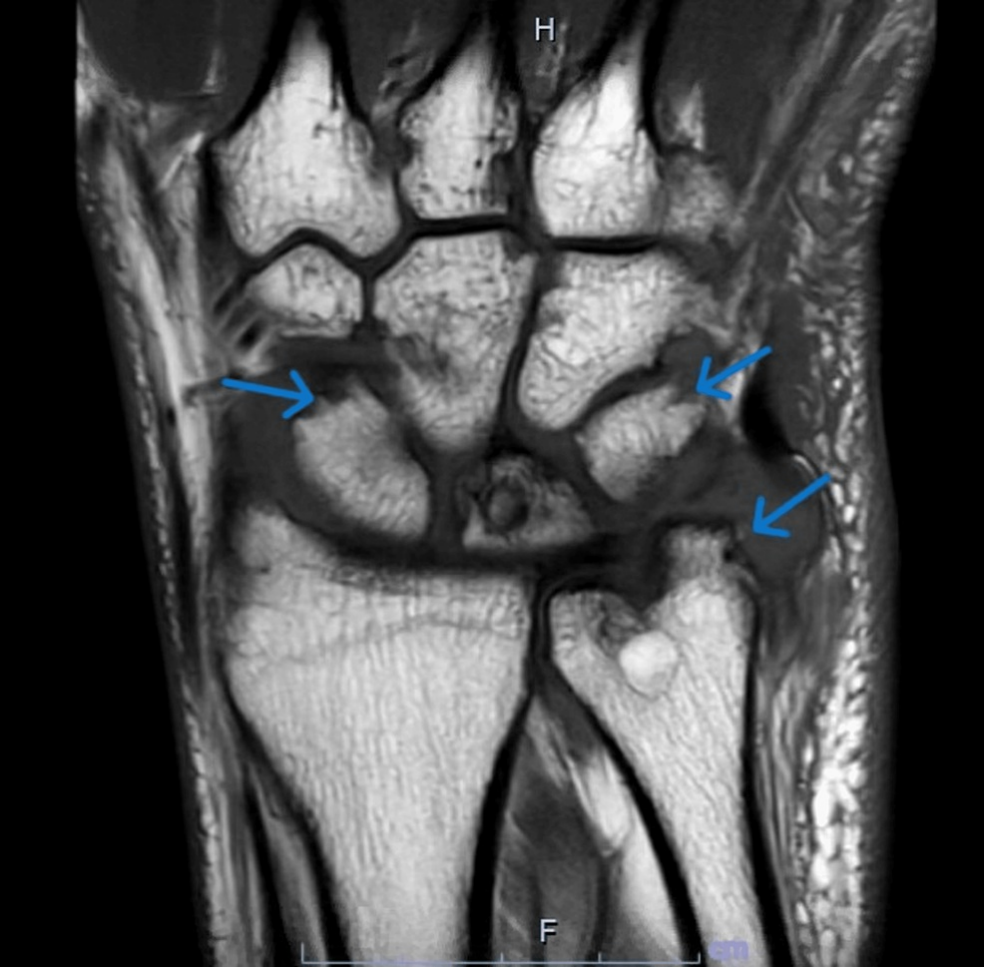
MRI of the right hand showing large periarticular erosions in the ulnar styloid, scaphoid, triquetrum, and capitate

X-rays of the bilateral wrists revealed diffuse narrowing of carpal joints with increased bony proliferative changes and erosions and soft tissue swelling at the ulnar styloid processes (Figure 1). Knee X-rays revealed mild bilateral patellofemoral joint space narrowing, periarticular osteophytes, and small suprapatellar effusions, right > left (Figure 2). X-rays of the feet revealed narrowing, sclerosis, and subchondral cyst at the right first PIP joint, and erosion of the bilateral second and third and the right first MTP joints (Figure 3). All X-ray findings were interpreted by a musculoskeletal radiologist as most consistent with a diagnosis of RA. As the right wrist joint was the most chronic and painful but without clinically visible effusion, an MRI was ordered. It revealed extensive periarticular erosions in the ulnar styloid, scaphoid, triquetrum, and capitate. Small joint effusion with synovitis was noted at the distal radioulnar, radiocarpal, and ulnocarpal joints. There was marked ulnar-sided and palmar non-enhancing soft tissue edema, which was again radiologically interpreted as inflammatory arthropathy, likely RA (Figure 4).

**Figure 5:**
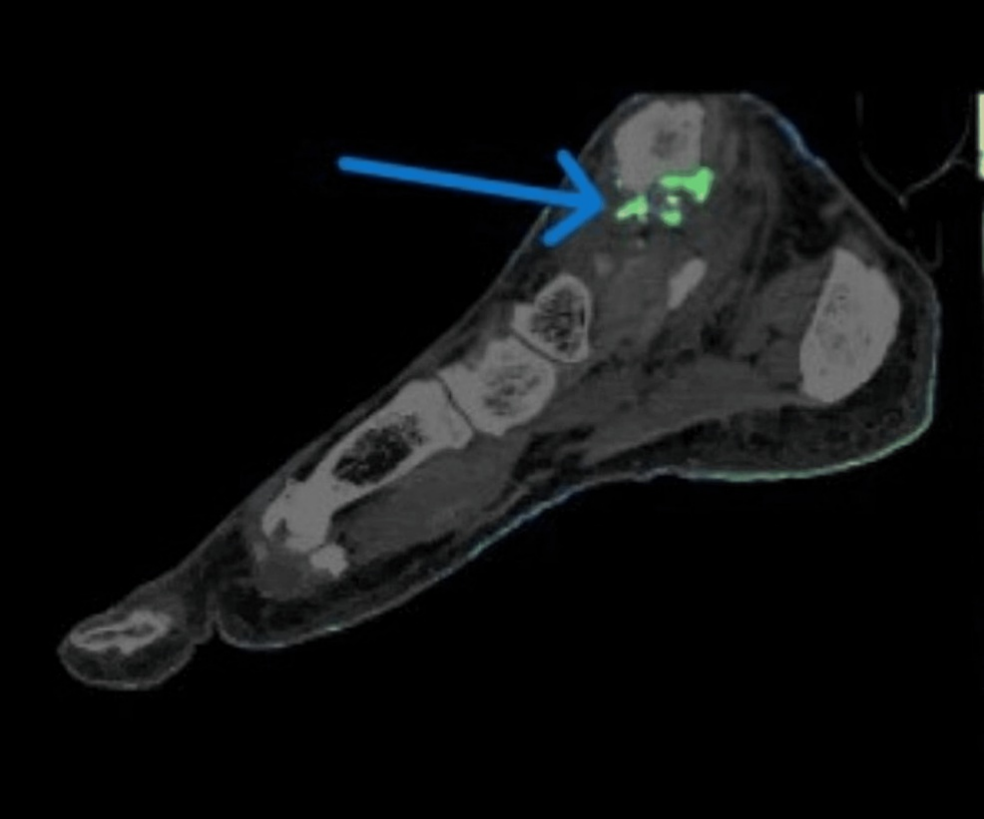
DECT of the right foot demonstrating uric acid deposition, most prominent in the region of the deltoid and posterior talofibular ligaments DECT: dual-energy computed tomography

**Figure 6:**
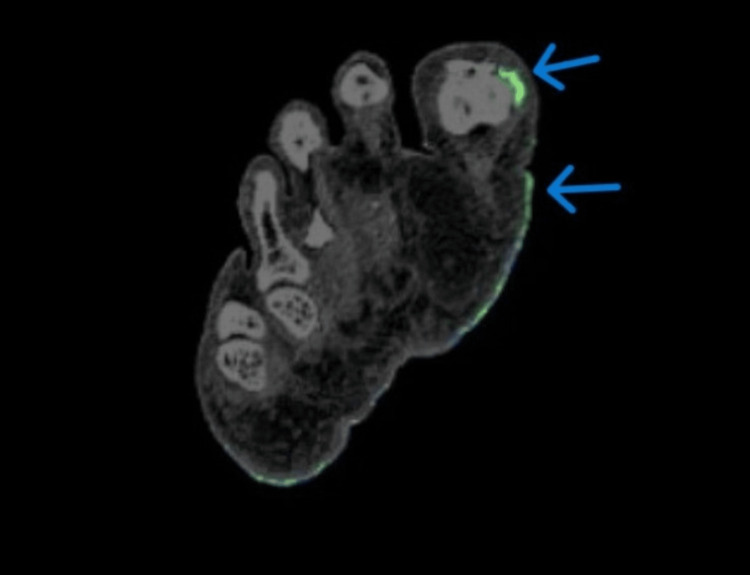
DECT of the right foot with uric acid deposition in the first MTP DECT: dual-energy computed tomography; MTP: metatarsophalangeal

**Figure 7:**
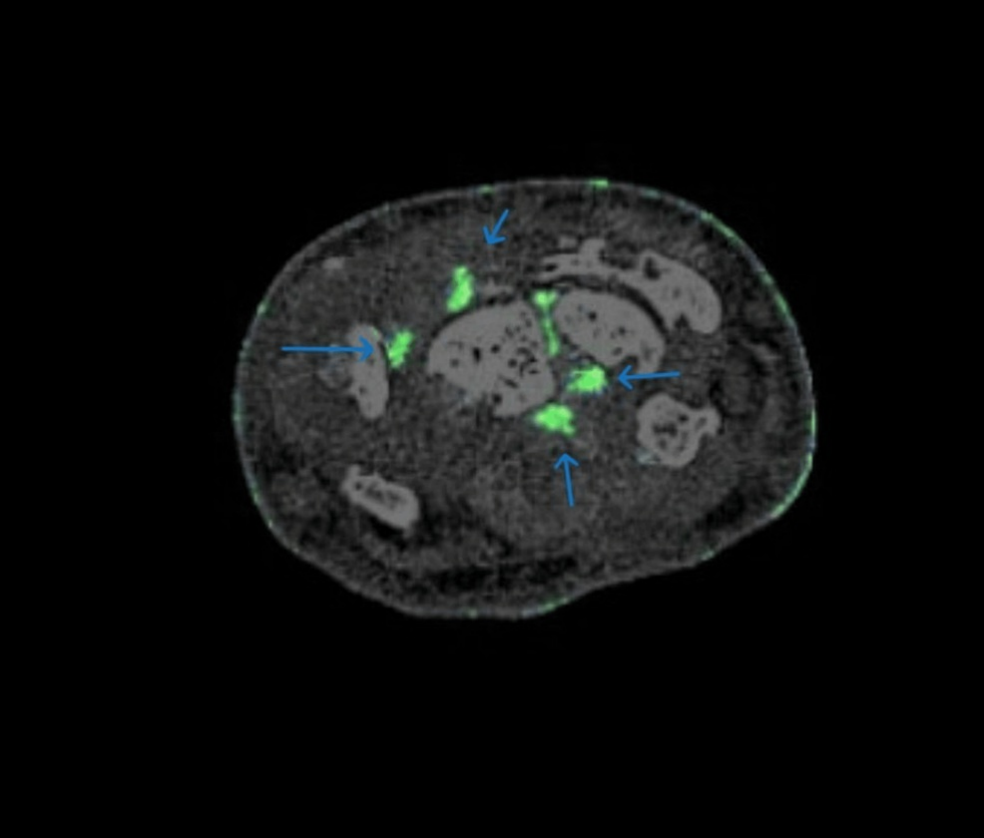
DECT of the right wrist showing uric acid deposition within the triangular fibrocartilage complex and scapholunate ligament DECT: dual-energy computed tomography

**Figure 8:**
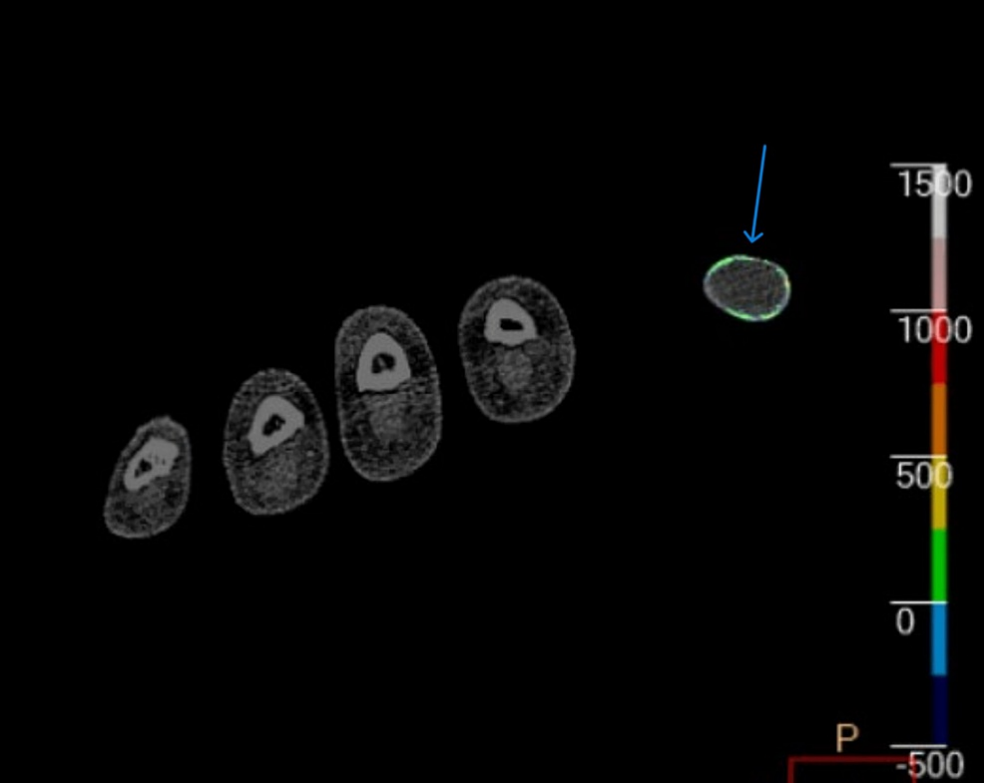
DECT revealing uric acid deposition and calcification within the flexor tendon at the level of the fifth proximal phalanx DECT: dual-energy computed tomography

**Figure 9:**
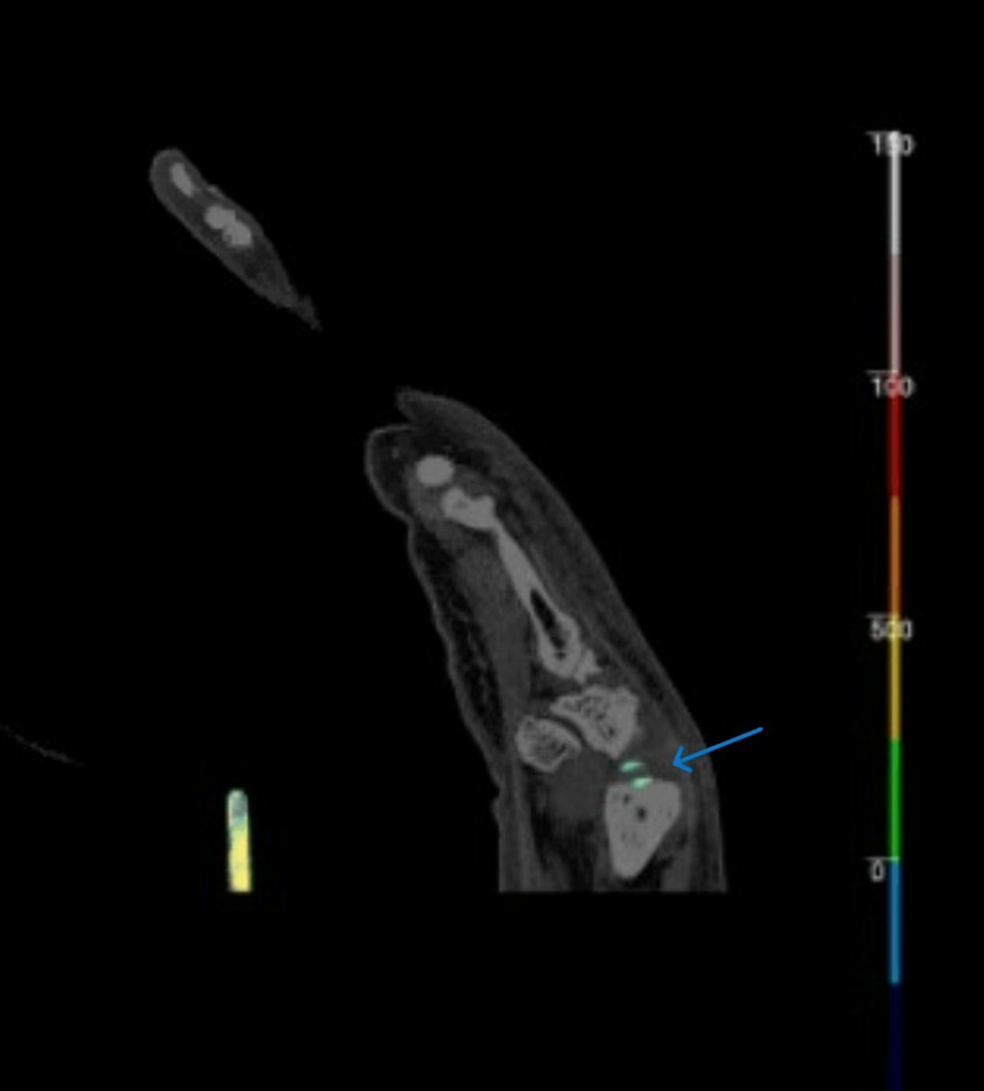
DECT showing uric acid deposition at the extensor tendon at the level of the fifth metacarpal head DECT: dual-energy computed tomography

Given the diagnostically confusing clinical and radiographic findings in multiple joints of the upper and lower extremities and raising the possibility of an alternative diagnosis, such as seronegative RA and no sizable joint effusion for aspiration, DECT of the right hand and right foot was ordered. The right foot DECT report noted a monosodium urate (MSU) deposition within the deltoid ligament, posterior talofibular ligament, Achilles tendon, flexor tendons of the hindfoot, second and third metatarsal heads, and talonavicular joints (Figures 5, 6). DECT of the right hand and wrist revealed MSU deposition at the triangular fibrocartilage complex, scapholunate ligament with periarticular erosions, flexor tendon of the fifth proximal phalanx, and extensor tendon of the fifth metacarpal head (Figures 7-9). The DECT report was interpreted as consistent with the diagnosis of polyarticular and periarticular gout. As the diagnosis of polyarticular gout was confirmed, the patient was advised to increase the allopurinol dose to 400 mg daily with target serum uric acid of <5 mg/dL, increase the colchicine dose to 0.6 mg BID, and change indomethacin from the as-needed regiment to 25 mg daily.

## Discussion

Gout is one of the most common inflammatory arthritis in the USA, with a prevalence of 3.9% and increasing. The most common initial presentation is a monoarticular rapid onset of pain, swelling, and redness [2]. Acute flares result from the deposition of MSU crystals in the joint(s) or periarticular structures, triggering an inflammatory response [3]. The first episode usually occurs after an asymptomatic period of hyperuricemia, lasts one to two weeks, and self-resolves [4]. The first MTP (90%), midfoot (25-50%), ankle (18-60%), knee, wrist, and fingers (6-25%) are affected in this order of decreasing frequency [5]. The periarticular structures like bursae, ligaments, and tendons may be affected, causing a diagnostic challenge as in our patient. The acute flare is usually monoarticular, but polyarticular flares can occur in patients with long-standing gout such as our patient [6]. Untreated or under-treated hyperuricemia in acute gout results in the evolution into subacute and chronic stages [7]. In chronic stages, the formation of tophi is common, typically after more than 10 years. Tophi are nodular masses formed by massive deposition of MSU crystals in the soft tissues, synovial tissues, and/or periarticular structures [8]. MSU crystals and tophi can cause extensive bone erosions leading to arthritis mimicking RA [8]. The pain becomes more constant and chronic rather than episodic and again mimics RA, as seen in our patient [7].

Some of the clinical features like the presence of tophi on physical examination but the absence of morning stiffness, symmetric joint involvement (especially of hands), and the lack of detection of serologic markers of RA are useful in distinguishing RA from polyarticular gout [7,8]. During the early stages of RA, there is synovitis and inflammation of the joints resulting in fusiform and symmetric juxta-articular soft tissue swelling [9]. This inflammation results in bone resorption and peri-articular osteopenia from demineralization [9]. These marginal erosions are the first erosive changes seen before the occurrence of joint space loss and narrowing [10]. In the wrist, erosive changes are typically seen in the ulnar styloid, waists of the scaphoid and hamate, as well as the fifth carpometacarpal joint, as seen in our patient. As the cartilage destruction continues, symmetric and uniform joint space narrowing becomes evident and there is the formation of subchondral erosions or cysts [10]. In gout, it usually takes an average of seven to 10 years for radiographic findings to appear. The imaging findings of gout, on the other hand, reveal an asymmetrical polyarticular distribution, juxta-articular eccentric, and lobulated soft tissue masses due to tophus deposition. There is normal mineralization so no osteopenia will be noted [11]. The erosions are well-defined with sclerotic margins that appear as punched-out lesions and overhanging margins, which are absent in this patient [11]. As synovitis is not the primary pathophysiology in gout, cartilage damage, and joint-space loss are not seen until later stages when secondary osteoarthritis develops. Extensive erosions from the long-standing soft-tissue tophi can give a characteristic “mouse or rat bite” appearance [11].

It is important to consider and rule out other types of inflammatory arthritides in patients presenting with polyarticular disease. The most common mimickers of chronic polyarticular gout include rheumatoid arthritis, psoriatic arthritis, and CPPD disease [12]. When there are overlapping clinical and radiographic findings, as in our case, DECT can be very helpful and should be performed. The sensitivity and specificity of arthrocentesis and synovial fluid analysis (SFA) are 84.4% and 97.2%, respectively [13]. A meta-analysis of patients diagnosed with gout via DECT shows DECT sensitivity and specificity closely approaching SFA at 81% and 91%, respectively [14,15]. Most importantly, DECT helps identify MSU crystals in extra-articular tissues that are difficult to access by arthrocentesis [16]. As seen in our patient, most MSU deposits on DECT are seen in periarticular structures, such as tendons and ligaments, and, therefore, are inaccessible by arthrocentesis. Of note, DECT can also identify calcium pyrophosphate crystals by their specific X-ray attenuation properties and help to differentiate gout from pseudogout/CPPD disease [17]. The latest 2015 American College of Rheumatology (ACR)/European Alliance of Associations for Rheumatology (EULAR) classification criteria for gout include criteria for positive DECT findings [18].

## Conclusions

In challenging cases with overlapping clinical and radiographic findings of inflammatory arthritides, including gout and rheumatoid arthritis, the use of DECT can facilitate reaching the correct diagnosis. This is especially true in cases with large MSU crystal deposition in tendons, ligaments, and bursae - areas difficult to access by arthrocentesis. DECT's high sensitivity and specificity make it a valuable diagnostic tool in such challenging cases.
